# Signal and Image Processing of Physiological Data: Methods for Diagnosis and Treatment Purposes

**DOI:** 10.1155/2016/1054605

**Published:** 2016-03-17

**Authors:** Anne Humeau-Heurtier, Edite Figueiras, Joao Cardoso

**Affiliations:** ^1^Laboratoire Angevin de Recherche en Ingénierie des Systèmes (LARIS), University of Angers, 62 avenue Notre-Dame du Lac, 49000 Angers, France; ^2^Ultrafast Bio- and Nanophotonics Group, International Iberian Nanotechnology Laboratory, Avenida Mestre José Veiga, 4715-330 Braga, Portugal; ^3^Department of Physics, LIBPhys-UC, University of Coimbra, Rua Larga, 3004-516 Coimbra, Portugal

Signals and images have taken a major place in clinical practice to assist medical experts in the decision-making process. They are now commonly used for research activities or in clinical routines, for the diagnosis or treatment of pathologies. However, this decision-making process requires the development of signal and image processing algorithms. This is why, in the clinical domain, signal and image processing algorithms aiming at extracting information, quantifying processes, or improving the visualization of medical data have exploded. New algorithms overcoming the existing ones by their better computational cost or by providing additional or more accurate information are still proposed. Moreover, new applications for the new and existing algorithms emerge. This is true for any kind of medical data but the physiological and pathophysiological questions offer a variety of applications and situations. This includes the study and understanding of adaptive responses in health and pathophysiological mechanisms in disease, at any level of physiological organization, ranging from molecules to humans. Moreover, adaptive, integrative, and translational physiology is concerned.

This special issue proposes different papers in this way of using computers to help in the diagnosis and in the therapy. Thus, for the field of medical computer vision, H. Li et al. propose a 3D facial feature point localization method that combines the relative angle histograms with multiscale constraints. Moreover, to help the medical staff in combining images coming from different modalities into one image, P. Geng et al. introduce a medical image fusion algorithm in spatial domain that uses an adaptive manifold filter. In the denoising field, L. Chang et al. propose a two-stage MRI denoising algorithm that is based on 3D optimized blockwise version of nonlocal means and multidimensional PCA. An insight into the relationship between the transient auditory-evoked potentials and synthetic steady-state responses at different stimulus rates under the superposition hypothesis is proposed by X. Tan et al., whereas M. Alvarado-González et al. introduce a study to describe a P300, an event-related potential endogenous component. A. Shakeel et al. have written a review of the features used for EEG data recording from different studies that used movement-related cortical potential to predict the upcoming real or imaginary movement. R. Faltermeier et al. propose an extension of a mathematical tool set, called selected correlation analysis, which detects positive and negative correlations between arterial blood pressure and intracranial pressure. This extension optimizes the parameters of the selected correlation analysis. U. Mäder et al. introduce an analysis procedure of digital microscopic images to increase diagnostic quality and to shorten the time-to-diagnosis for superficial fungal infections. F. Espinoza-Cuadros et al. investigate the use of both patients' facial images and voice recordings processing to estimate the apnea-hypopnea index that describes the severity of the obstructive sleep apnea. S. Tagliaferri et al. use acceleration and deceleration phase rectified slope of computerized cardiotocographic traces for the identification and management of intrauterine growth restriction of fetuses and in the prediction of the neonatal outcome. In the field of laryngeal functionality, using endoscopic images, S. Petermann et al. investigated analytical approaches for computing the voice onset time. Finally, M. Zhu et al. introduce an improved niche genetic algorithm for feature reduction in high-dimensional data.

The wide biomedical engineering community (both medical and scientists communities) is expected to be interested by this special issue. Both researchers and practitioners will find results to go ahead in the diagnosis and treatment of pathologies.

